# Optimized Liquid-Liquid Extractive Rerefining of Spent Lubricants

**DOI:** 10.1155/2014/458789

**Published:** 2014-02-12

**Authors:** Muhammad Ashraf Kamal, Syed Mumtaz Danish Naqvi, Fasihullah Khan

**Affiliations:** ^1^Department of Applied Chemistry and Chemical Technology, University of Karachi, Karachi 75270, Pakistan; ^2^Department of Chemical Engineering, University of Karachi, Karachi 75270, Pakistan

## Abstract

Central composite design methodology has been employed to model the sludge yield data obtained during liquid-liquid extractive rerefining of spent lubricants using an alcohol (1-butanol) and a ketone (methyl ethyl ketone) as prospective solvents. The study has resulted in two reasonably accurate multivariate process models that relate the sludge yield (*R* ^2^ = 0.9065 and 0.9072 for alcohol and ketone, resp.) to process variables (settling time *t*, operating temperature *T*, and oil to solvent ratio *r*). Construction of such models has allowed the maximization of the sludge yield (more than 8% and 3% in case of alcohol and ketone, resp.) so that the extraction of useable oil components from spent lubricants can economically be performed under extremely mild conditions (*t* = 16.7 h, *T* = 10°C, and *r* = 2) and fairly moderate conditions (*t* = 26.6 h, *T* = 10°C, and *r* = 5) established for the alcohol and ketone correspondingly. Based on these performance parameters alcohol appears to be superior over ketone for this extraction process. Additionally extractive treatment results in oil stocks with lesser quantity of environmentally hazardous polyaromatic hydrocarbons that are largely left in the separated sludge.

## 1. Introduction

In this modern technological society, the increasing number of power generating systems, for various services, requires ever rising volumes of lubricants. During usage lubricants are exposed to severe conditions resulting in an extremely complex mixture containing unaltered useable oil components along with carbon residue, hetrocompounds, heavy metals, and especially poly-aromatic hydrocarbons (PAH) that are recognized potent carcinogens and mutagens [1–3]. Untreated disposal of spent lubricants therefore poses serious environmental hazards particularly due to the presence of considerable quantities of PAH and has therefore evolved as a serious global environmental issue. On the other hand, spent lubricants also contain substantially high proportions of precious unaltered lube base oil components that by proper treatment can be recovered for reuse during blending of fresh lubricants.

Rerefining of spent lubricants therefore should have been associated with two major objectives, that is, recovery of useable components and proper disposal of environmentally hazardous materials. Although various processes have been described in the literature based on liquid-liquid extraction of useable oil components and separation of the perilous substances as sludge [4–8] but none of them describe any optimized technique for economical rerefining of spent lubricants.

This novel work describes a purely quantitative treatment of the liquid-liquid extraction of the useable oil component from spent lubricants. It consists of the construction of process models based on central composite design (CCD) to simulate the sludge yield during liquid-liquid extraction of spent lubricants. CCD approach is a technique for the response surface study that involves modeling the relationship between quantitative process variables and the response variables and locating the combination of the process variable that gives the optimum expected response [9–15].

Briefly this research consists of the investigation of the quantitative effect of process variables (settling time *t*, operating temperature *T*, and oil to solvent ratio *r*) upon liquid-liquid extraction of useable components of spent lubricants with a representative alcohol (1-butanol) and a ketone (methyl ethyl ketone) commonly used as solvents for such extractive rerefining [4–8]. The novel purpose behind the present study was to establish the appropriateness of either solvent in a statistically significant manner in order to prescribe the optimum solvent and operating conditions for maximum removal of obnoxious components of spent lubricant in the form of sludge. Consequently such optimum conditions will be responsible for maximum sludge yield. Since the spent lubricants sludge is mainly composed of resinous substances which are largely poly-aromatic systems; therefore, the larger the sludge yield the larger the decrease in the PAH content of the extracted lubricants that is in accordance with the requirements of the environmental protection agencies.

## 2. Methodology

### 2.1. Multivariate Process Models for Sludge Yield

**Figure 1:**
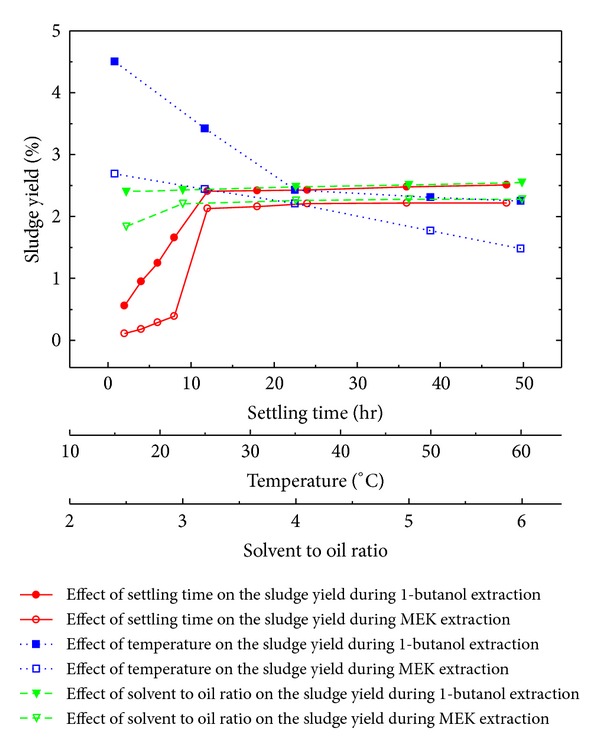
Experimental sludge yield during liquid-liquid solvent extraction of a representative spent lubricant as function of process variables *t*, *T*, and *r*.

**Figure 2:**
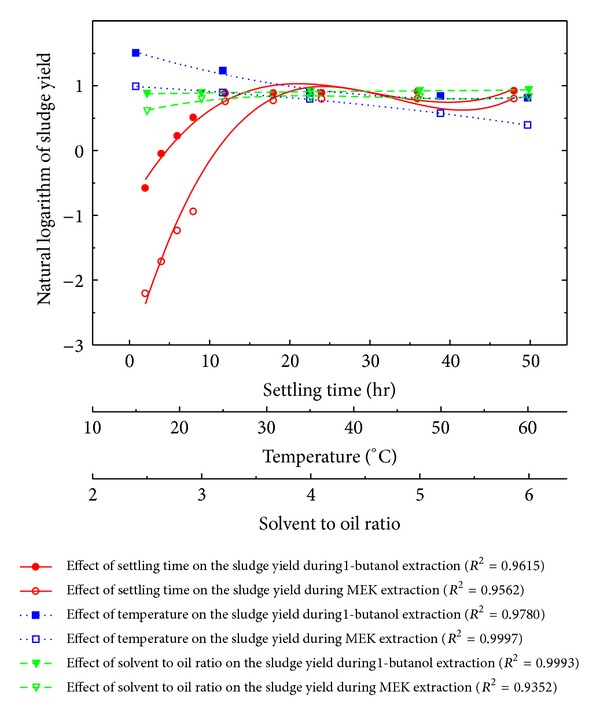
Cubic functional relations between sludge yield and process variables *t*, *T*, and *r* during liquid-liquid extraction.

The sludge yield *Y* (*Y*_*A*_ and *Y*_*K*_ in case of alcohol and ketone, resp.) during spent lubricants extraction process varies with the process variables *χ* (*χ*_1_ = *t*, *χ*_2_ = *T*, *χ*_3_ = *r*). Preliminary experimentation reveals that such effects are nonlinear (Figure 1) and it has been discovered during present study that cubic polynomial functions excellently describe these relations, if natural logarithm of sludge yield is used as ordinate (Figure 2). Further, simultaneous variation in ln⁡(*Y*) against process variables may be described by a multivariate cubic polynomial (MCP). This MCP of three variables *χ* contains, in addition to an intercept and nine power terms, every possible two and three parameter interactions, making *N* = 20 predictor variables (PVs), which could be the mathematical model of choice for this system:

(1) ln⁡(Y)=β0+β1χ1+β2χ12+β3χ13+β4χ2+β5χ22+β6χ23 +β7χ3+β8χ32+β9χ33+∑i=10N−1βi∏j=13χjγj,

where *β*_*i*_ are the coefficients of MCP and the last term represents the summation over all the possible two and three parameter interactions with *γ* = 0,…, 2, where *γ* is the power of *χ* in the interaction terms.

This model contains the transformed ordinate ln⁡(*Y*) instead of the original response *Y*. Transformation of response is quite normal during development of process models. Such transformations usually lead to simpler models with better fitting capabilities. Moreover, the models with transformed response provide better variance stabilization and simple interpretation of the effects of PVs and consequently result in the most parsimonious model with the fewest PVs [9].

### 2.2. Design Matrix and Response Vectors

CCD is usually employed to study and optimize processes and the same has been used here in order to maximize the sludge yield (target response during liquid-liquid extraction of spent lubricant that affect the quality of re-refined lube base oil) against process variables *χ*. The CCD has been constructed by stacking nine rows for partial factorial design of three variables at three levels, seven rows of star design at three levels, and six replicates in the center making a total of twenty-two (*M* = 22) experiments. Partial factorial design was chosen to increase the number of levels without having excessive number of experiments, as is usual with the multilevel full factorial designs. At the same time it provides a statistically significant orthogonal experimental design. A full factorial design of three variables at three levels would contain twenty-seven experiments that have significantly reduced to just nine experiments in the case of partial factorial design. If full factorial design was selected, it would result in a CCD having forty experiments. These extra experiments do not always result in useful extra information and so are wastage of time and resources. The use of partial factorial design has therefore reduced the total number of experiments to almost half as would otherwise be required [10]. If *y* = ln⁡(*Y*), *x*_1_ = *χ*_1_, *x*_2_ = *χ*_2_, *x*_3_ = *χ*_3_, *x*_4_ = *χ*_1_^2^, and so on, (1) may simply be written as

(2) $$\begin{array}{c} y=\beta_{0}+\sum_{i=1}^{N-1}\beta_{i}x_{i}. \end{array}$$

In this manner the design matrix **X**(*M* × *N*) and the response vectors **y**_**A**_(*M* × 1) and **y**_**K**_(*M* × 1) have been constructed, in accordance with (2).

(3) X=[1x11⋯x1N⋮⋮⋱⋮1xM1⋯xMN],  yA=[ln⁡(YA1)⋮ln⁡(YAM)]

(4) $$\begin{array}{c} y_{K}=\left[\begin{array}{c} \ln\left(Y_{K1}\right)\\ \vdots \\ \ln\left(Y_{KM}\right) \end{array}\right]. \end{array}$$

This arrangement contains sufficient (*M* > *N*) data points, for assessment of the statistical significance of the resulting models. Before further processing, matrix **X** and vectors **y**_**A**_ and **y**_**K**_ have separately been simultaneously sorted according to *Y*_*A*_ or *Y*_*K*_ being in ascending order. The sorted CCDs and response data for liquid-liquid extraction of spent lubricant may be seen in Tables 1 and 2.

### 2.3. Multiple Linear Regression (MLR)

As (2) is linear in all the coefficients *β*_*i*_, the coefficients can be estimated by the procedure of MLR, which results in the following equations for the estimates of *β*_*i*_ [9–12]:

(5) $$\begin{array}{c} {\hat{\beta }}_{A}={\left(X^{T}X\right)}^{-1}X^{T}y_{A},\\ {\hat{\beta }}_{K}={\left(X^{T}X\right)}^{-1}X^{T}y_{K}, \end{array}$$

where ${\hat{\beta }}_{A}(N\times 1)$ or ${\hat{\beta }}_{K}(N\times 1)$ are the vectors of the estimates of the coefficients *β*_*i*_.

The backward elimination variable selection technique has been employed in the present study to develop the multivariate process models for the sludge yield during liquid-liquid extraction of spent lubricant. The indicators for the goodness of fit, during MLR, were the coefficients of determination *R* ^2^ or *R*_Adjusted_^2^ defined as [12, 13]

(6) $$\begin{array}{c} R^{2}=\frac{\text{S}{\text{S}}_{\text{Regression}}}{\text{S}{\text{S}}_{\text{Total}}},\;\;R_{\text{Adjusted}}^{2}=1-\frac{\text{S}{\text{S}}_{\text{Residual}}/\text{D}{\text{F}}_{\text{Residual}}}{\text{S}{\text{S}}_{\text{Total}}/\text{D}{\text{F}}_{\text{Total}}}. \end{array}$$

### 2.4. Optimization

The solvent extraction of useable components of spent lubricants results in the settling of insoluble substances in the form of sludge. The more the sludge formed during this extractive treatment, the more refined the resulting lube base oil devoid of significant PAH and the more economical the process would be. Therefore, one of the key goals during the present study was to maximize the sludge yield using two prospective solvents. The optimization results in the specification of optimum operating condition for maximum sludge yield.

## 3. Experimental Section

The experimental procedures for solvent extraction of spent lubricants, thin layer chromatographic (TLC) analyses, and ultraviolet (UV) analyses were described elsewhere [7, 16–18]. However, appropriate modifications in the UV analyses have been adapted. UV analyses of samples were performed on Shimadzu UV-1601PC double beam spectrophotometer in matched 1.0 cm silica cells against pure solvent. Samples (approximately 0.02 g) were accurately weighed to the nearest 0.1 mg into 10 mL volumetric flasks and diluted with HPLC grade isooctane. Resulting solutions were further diluted by a factor of 1/100 with the same solvent. Scanning parameters: wavelength = 225–400 nm, scan speed = 200 nm/min, spectral band width = 1.0 nm, sampling interval = 0.2 nm, and recording range = 0.0–0.4. Spectra were normalized by multiplying absorbance readings by 0.02/*w*_Oil_.

All the mathematical results presented herein were obtained using Mathcad Professional (Math Soft, Inc.) and Microsoft Excel (Microsoft Corporation). Modeling of sludge yield data have resulted in two objective functions, (7) and (8), for the extraction with alcohol and ketone, respectively. The optimization solver of Mathcad had automatically selected conjugate gradient method (CGM) for the solution. The optimization process was subjected to the constraints, 10 ≤ *χ*_1_ ≤ 50 h, 10 ≤ *χ*_2_ ≤ 60°C, and 2 ≤ *χ*_3_ ≤ 6.

## 4. Results and Discussion

The modeling in either case (models for *Y*_*A*_ and *Y*_*K*_) was started using the full model (2). It was desired to cautiously arrive at the best possible models containing the smallest number of PVs that best describe the variation of the sludge yield against process variables *χ* in a statistically significant manner. As described in Section 2.3., these determinations of the parsimonious models have been achieved using backward elimination technique consisting of eliminating all the nonsignificant PVs associated with a *P* value larger than 0.05. As a result the following two multivariate process models have been resulted:

(7) ln⁡(YA)=β0χ1+β1χ12+β2χ13+β3χ2+β4χ32+β5χ33 +β6χ1χ2+β7χ1χ3+β8χ1χ32,

(8) ln⁡(YK)=β0χ1+β1χ12+β2χ32+β3χ33+β4χ1χ3 +β5χ2χ3+β6χ12x3+β7χ2χ32.

The final regression results (Table 3) include the estimated coefficients *β*_*i*_, *t* values, and *P*-values associated with each PV in the parsimonious process models represented by (7) and (8).

### 4.1. Process Model for *Y*_*A*_

**Figure 3:**
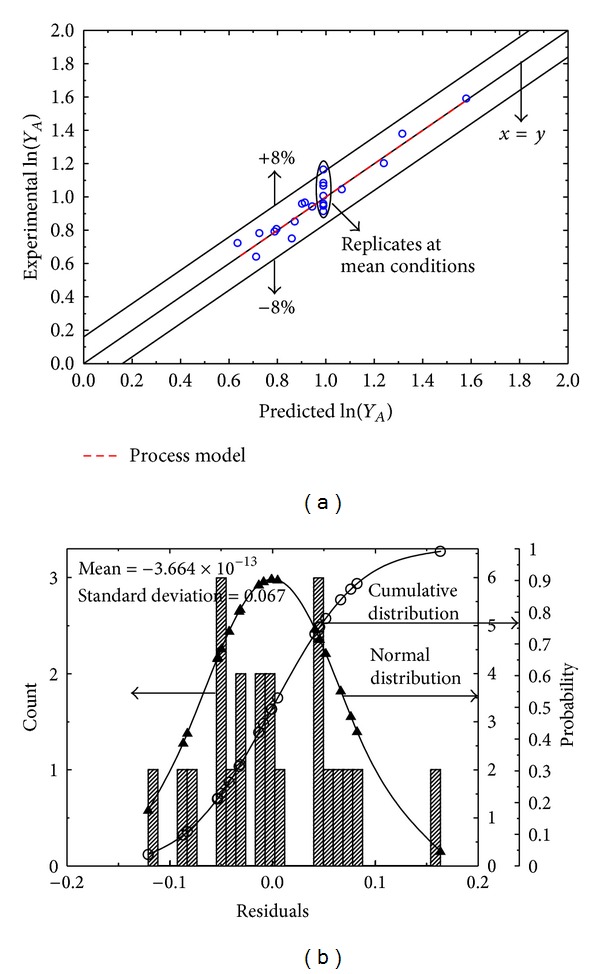
(a) Multivariate model relating the sludge yield *Y*_*A*_ during liquid-liquid extraction with alcohol to process variables *t*, *T*, and *r*. (b) Corresponding residual analysis.

The statistical details of the process model for *Y*_*A*_ can be found in Table 4 and Figure 3. In the analysis of variance Table 4 the amount of variability explained by MLR (0.1136) is sufficiently greater than the amount due to residual error (0.0072). The difference is large enough (*P* value is close to 0) to strongly reject the null hypothesis, that is, no MLR relationship exists between ln⁡(*Y*_*A*_) and the retained PVs. The value, *R* ^2^ = 0.9065, indicates that more than 90% of the variation in the calibration response data has been absorbed by the process model. Furthermore the residuals follow the classical normal distribution around an essentially zero mean (−3.664 × 10^−13^) along with a sufficiently small standard deviation of 0.067 (Figure 3(b)).

The process model represented by the dashed red line actually overlaps the black solid hairline for ideal fit (*x* = *y*) indicating the high quality of fit of the response data (Figure 3(a)). Moreover the prediction for calibration samples just spans over a favorably narrow range of ±8% around the line for ideal fit. The model is associated with a sufficiently low (0.0343) mean square replicate error (Table 4) and the replicates are evenly distributed around the line of best fit (Figure 3(a)). As far as the *F* ratio of mean square lack-of-fit to replicate error is concerned, it is tolerably close to unity, suggesting both errors are of the same order of magnitude and therefore the model describes the sludge yield data significantly well. These are strong evidences, ensuring that in new circumstances (within process variable constraints) the process model will have good predictive capability for *Y*_*A*_.

The process model (7) contains the most significant PVs associated with reasonably large absolute *t* values (>2) with corresponding *P* values much smaller than 0.05 in most of the cases (Table 3). Therefore all the retained variables in the process model are statistically significant although relative significances may be more or less based on the magnitudes of the *t* values. The significant PVs in the process model include linear (*χ*_1_ and *χ*_2_), quadratic (*χ*_1_^2^ and *χ*_3_^2^), cubic (*χ*_1_^3^ and *χ*_3_^3^), and interaction (*χ*_1_ *χ*_2_, *χ*_1_ *χ*_3_, and *χ*_1_ *χ*_3_^2^) terms.

The PVs having the strongest effect on ln⁡(*Y*_*A*_) are *χ*_1_, *χ*_1_^2^, and *χ*_1_^3^ as these are associated with comparatively much higher *t* values among the significant PVs and have got zero *P* values, suggesting their highly significant nature (Table 3). Among these PVs *χ*_1_ has got the largest *t* value and a positive sign for its coefficient indicates direct relation between settling time and sludge yield during liquid-liquid extraction of spent lubricant with alcohol. However, the relation between *χ*_1_ and ln⁡(*Y*_*A*_) was assumed to be nonlinear (Figure 2). That intricate nonlinear nature is reflected by the retained terms *χ*_1_^2^ and *χ*_1_^3^, where the fine adjustment is being made by these terms. In this manner settling time has emerged as the most important process variable that dictates the sludge yield during liquid-liquid extraction of spent lubricant with the representative alcohol. The next most significant factor that greatly influences the sludge yield is *χ*_3_^2^, as it is associated with a quite higher *t* value and an almost zero *P* value although the effect is nonlinear. Fine tuning in nonlinear nature of the effect is contributed by *χ*_3_^3^. That supports the hypothesis that cubic polynomial relations exist between ln⁡(*Y*_*A*_) and *χ*_1_ and *χ*_3_.

On the other hand, the negative sign for the coefficient for *χ*_2_ is in accordance with Figure 3, where it can be noticed that there is an inverse relation between temperature and ln⁡(*Y*_*A*_) that can be well approximated by a straight line (*R* ^2^ = 0.8263). Small negative slope (−0.0151) of such a straight line is close to the coefficient for *χ*_2_(−0.0119). Therefore, it has been established that temperature does not play any significant role during extraction with alcohol. The relative insignificance of *χ*_2_ as compared to *χ*_1_ can also be deduced from a higher *P* value. However, the mutual effect of *χ*_1_ and *χ*_2_ represented by the retained interaction term *χ*_1_ *χ*_2_ has some significance on the response as can be noticed in the response surface diagram (Figure 5(a1)). Here shorter settling time and low temperature maximize the sludge yield during extraction with alcohol.

Hitherto it has become clear that the two most important process variables that greatly influence the sludge yield during extraction of spent lubricant with alcohol are the settling time *χ*_1_ and the solvent to oil ratio *χ*_3_. The combined effect of *χ*_1_ and *χ*_3_ has been captured by the nonlinear interaction terms *χ*_1_ *χ*_3_, and *χ*_1_ *χ*_3_^2^. The influential nature of these two process variables can be seen in the response surface diagram (Figure 5(b1)). This diagram shows that shorter settling time along with lower solvent to oil ratio will simultaneously be responsible for higher sludge yield. This diagram also presents the quality of prediction of the process model for *Y*_*A*_ as the response surface predicted by the process model (dotted mesh) has excellently been overlaid on the surface created using the experimental sludge yield calibration data (color map) contained in Table 1.

### 4.2. Process Model for *Y*_*K*_

**Figure 4:**
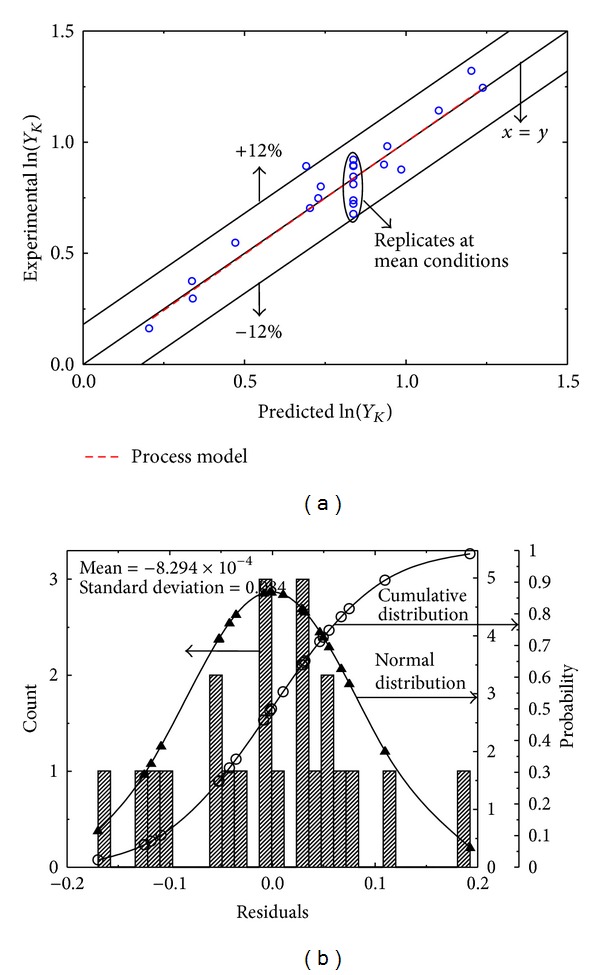
(a) Multivariate model relating the sludge yield *Y*_*K*_ during liquid-liquid extraction with ketone to process variables *t*, *T*, and *r*. (b) Corresponding residual analysis.

The quality of the process model for sludge yield during extraction with ketone can be noticed in Table 4 and Figure 4. Here the residuals once again follow the classical normal distribution around an approximately zero mean (−8.294 × 10^−4^) along with a slightly higher standard deviation of 0.084. The model is associated with comparable *R* ^2^ = 0.9072, indicating quite high quality of fit for the calibration response data. An excellent overlap of the process model (red dashed line) onto the line for ideal fit (*x* = *y*) again indicates the true capture, by the process model, of the precise variation in ln⁡(*Y*_*K*_) against process variables. However, reliability of the predictions is not as comparable as it is associated with a slightly higher prediction bounds of ±12% around the line for ideal fit. On the other hand this process model is again associated with the similar *F* ratio of mean square lack-of-fit to replicate error so that the model again has reasonable ability to represent the sludge yield data during extraction of spent lubricant with ketone.

Settling time *χ*_1_ has once again emerged as the most dominant process variable (Table 3). However, the relation between *χ*_1_ and ln⁡(*Y*_*K*_) is not linear and in fact there exists a quadratic relation between these quantities. The coefficient for *χ*_1_ is associated with the largest absolute *t* value along with a zero *P* value whereas *χ*_1_^2^ linked to a significantly large *t* value and an almost zero *P* value. Likewise, *χ*_3_^2^ and *χ*_3_^3^ are found to be the next most important PVs describing the nonlinear relation between the response and the solvent to oil ratio. The joint nonlinear effect of these two important process variables is captured by the interaction terms *χ*_1_ *χ*_3_ and *χ*_1_^2^ *χ*_3_. Yet again this model suggests that temperature *χ*_2_ has no direct impact on the sludge yield. It only enters into the model as nonlinear interaction terms *χ*_2_ *χ*_3_ and *χ*_2_ *χ*_3_^2^.

**Figure 5:**
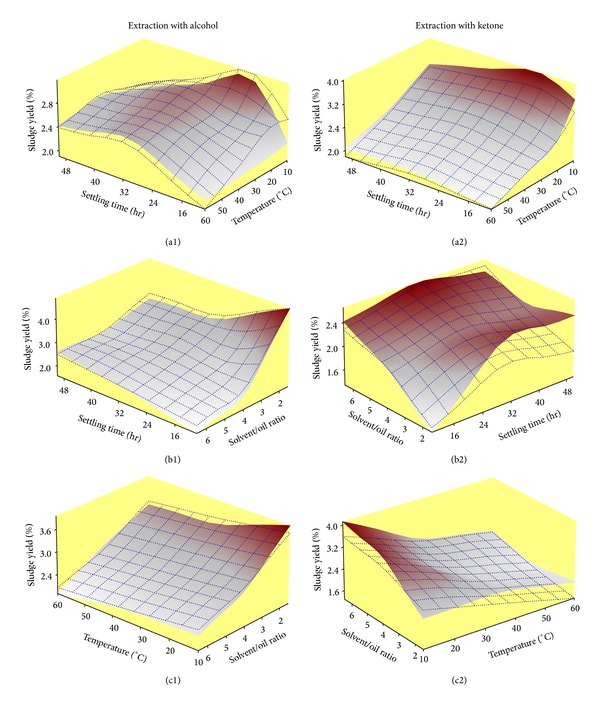
(a) Comparison of response surfaces describing simultaneous variation of sludge yield against *t* and *T*. (b) Against *t* and *r*. (c) Against *T* and *r*. Color maps are actual response surfaces while superimposed dotted mesh are the response surfaces, predicted by the multivariate process models (7) or (8).

The simultaneous effect of process variables upon sludge yield during extraction with ketone can be seen in the response surface diagrams (Figures 5(a2), 5(b2), and 5(c2)). These diagrams show that intermediate settling time at low temperature and high solvent to oil ratio will be responsible for the maximization of sludge yield during extraction with ketone.

### 4.3. Optimized Extraction Responses and Experimental Validation

The two objective functions (7) and (8) allow the sludge yield during liquid-liquid extraction of spent lubricant to be maximized. This process of optimization (subject to constraints described in Section 3) consists of finding the direction in which process variables *χ* should be varied in order to maximize the sludge yield. The working conditions for maximization of sludge yield in case of extraction of spent lubricant with alcohol have been determined as *t* = 16.7 h, *T* = 10°C, and *r* = 2. On the other hand, in case of extraction with ketone such conditions are *t* = 26.6 h, *T* = 10°C, and *r* = 4.99. These optimum working conditions are in excellent agreement with the response surface diagrams (Figure 5).

Extra experiments had been performed in order to test the validity of the prescribed optimum working conditions. These results are summarized in Table 5. There is a remarkable similarity present between predicted and experimental sludge yields with absolute relative errors well under 10%. This validation establishes the quality of predictions of the process models in the new circumstances within the experimental ranges for the process variables used during investigation.

This validation analysis finally proves on the statistical ground that the extraction with alcohol is preferable over ketone as it involves almost 60% lower solvent to oil ratio and 37% shorter settling time. At the same time it is responsible for 61% higher sludge yield as compared to extraction with ketone.

### 4.4. Quality Comparison of Oils Extracted at Optimized Conditions

**Figure 6:**
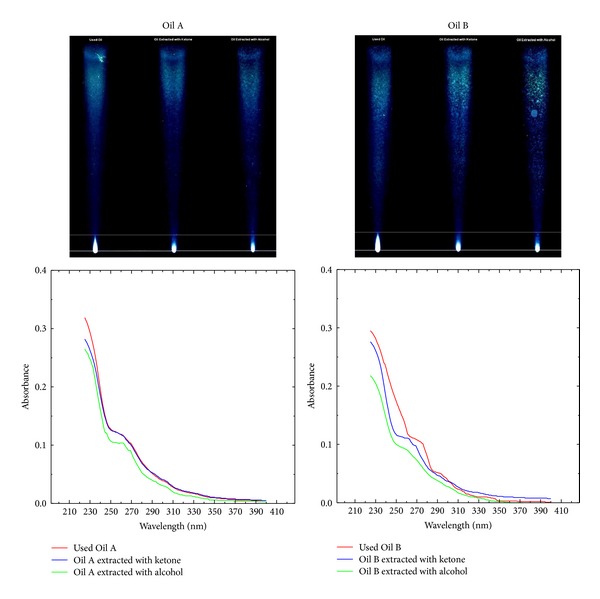
Processed images of the TLC plates developed for the comparative analyses of two lubricant samples along with the corresponding UV analyses illustrating the effect of the removal of sludge containing PAH upon extraction of spent lubricant samples with ketone and alcohol using corresponding optimum extraction parameters for the two solvents.

For quality assessment in terms of the PAH contents, two spent lubricant samples (designated as Oil A and Oil B) and the corresponding extracted oils (under optimized conditions) were subjected to TLC and UV analyses. The comparative TLC profiles (Figure 6) are continuous tailing bands in which separation of various compound types is quite distinct although overlapping behavior is present throughout. Various bands of different color intensity in the TLC profiles may be identified on the basis of the retardation factor [19]. The TLC profiles clearly indicate that saturates form the major portion of the spent and extracted lubricants that are present around the top of the profiles. Whereas, small amounts of highly adsorbing higher PAH and hetrocompounds are present near the bottom in the form of bright spurts. However, for the extracted oils such symmetrical spurts get diminished and rather diffused, indicating comparatively lower amounts of PAH and hetrocompounds. In between saturates and higher PAH there exists a series of lower PAH.

These observations have been supported by the UV analyses of oils that are particularly suited to reveal the nature of the aromatic portion of the oils consisting of conjugated unsaturated systems [17–19]. The acquired UV spectra of the oils are spectroscopic overlaps of the spectra of hundreds of conjugated unsaturated compounds having UV absorptivities, for example, PAH and hetrocompounds. As for very dilute solutions, Beer's law holds whereby absorbance is proportional to the concentration of absorbing species, so UV spectra under consideration may also be used to deduce quantitative information, such as relative abundance of compound types. In the selected spectral region saturates and olefins do not interfere as they usually have absorption maxima below 200 nm. Maxima in the spectra lie around 230 nm a common wavelength for diaromatics in oils whereas higher PAH may be seen around 254 nm [17, 18, 20]. The progressive decrease in PAH as a result of extractive rerefining may clearly be noticed at 254 nm. Categorically alcohol again proves to be a better solvent for such extractive prerefining as it has somewhat greater ability to reduce the PAH as can be seen in the comparative UV spectra.

## 5. Conclusion

Liquid-liquid extraction has been proved, in a statistically significant manner, an extremely efficient rerefining technique for spent lubricants. It has been statistically as well as categorically established that such extraction process using alcohol as the solvent involves milder process conditions and at the same time responsible for higher sludge yield (>8%) and consequently responsible for better removal of PAH. Such optimized mild conditions could have a direct impact on the economics of the process as it does not involve the wasteful of time and resources. In essence this optimized extractive rerefining of spent lubricants could be the process of choice to produce better quality feed for the subsequent blending or further refining to get superior lube oil.

## Figures and Tables

**Table 1:** Sorted CCD and response data for liquid-liquid extraction of spent lubricant with alcohol (1-butanol).

Run	Process variables			Response	
	t (h)	T (°C)	r	Y_A_ (%)	ln⁡(Y_A_)
1	30	60	6	1.8868	0.6349
2	10	60	4	1.0476	0.7167
3	10	35	4	1.1041	0.7439
4	10	10	6	1.1727	0.7760
5	30	35	6	1.1935	0.7855
6	50	60	2	1.2272	0.8007
7	50	10	4	1.3295	0.8457
8	30	35	4	1.4789	0.9078
9	50	35	4	1.5488	0.9356
10	30	35	4	1.5627	0.9411
11	30	35	4	1.5641	0.9416
12	30	35	4	1.5924	0.9526
13	50	35	6	1.5932	0.9529
14	30	60	4	1.6111	0.9598
15	30	35	4	1.7177	0.9998
16	30	10	4	1.8255	1.0387
17	30	35	4	1.8899	1.0612
18	30	35	4	1.9362	1.0771
19	30	35	4	3.1844	1.1583
20	30	35	2	3.3053	1.1955
21	30	10	2	3.9488	1.3734
22	10	35	2	4.8753	1.5842

**Table 2:** Sorted CCD and response data for liquid-liquid extraction of spent lubricant with ketone (methyl ethyl ketone).

Run	Process variables			Response	
	*t* (h)	*T* (°C)	*r*	*Y*_*K*_ (%)	ln⁡(Y_K_)
1	50	60	2	1.1699	0.1569
2	10	35	2	1.3385	0.2915
3	10	60	4	1.4477	0.3700
4	30	60	4	1.7203	0.5425
5	30	35	4	1.9575	0.6717
6	10	35	4	1.0101	0.6982
7	30	35	4	1.0476	0.7167
8	30	35	4	1.0811	0.7329
9	30	60	6	1.1018	0.7428
10	50	35	4	1.2148	0.7952
11	30	35	4	1.2384	0.8058
12	30	35	4	1.3168	0.8402
13	30	10	2	1.3902	0.8714
14	30	35	2	1.4280	0.8871
15	30	35	4	1.4283	0.8872
16	30	35	4	1.4363	0.8905
17	50	35	6	1.4458	0.8944
18	30	35	4	1.4986	0.9157
19	30	35	6	1.6554	0.9766
20	50	10	4	3.1180	1.1372
21	10	10	6	3.4540	1.2395
22	30	10	4	3.7284	1.3160

**Table 3:** Coefficients and their significance in the multivariate process models for the sludge yields during liquid-liquid extraction.

Model for ln⁡(*Y*_*A*_) (7)				Model for ln⁡(*Y*_*K*_) (8)			
*i*	*β*_*i*_	*t* value	*P* value	*i*	*β*_*i*_	*t* value	*P* value
0	3.4647152913*E* − 1	14.0289	0	0	7.0973841576*E* − 2	5.9947	0
1	−1.6031743008*E* − 2	11.2060	0	1	−1.0642033303*E* − 3	4.5225	0.0005
2	1.7569070873*E* − 4	10.8303	0	2	1.2171694089*E* − 1	5.7975	0
3	−1.1948361975*E* − 2	3.2110	0.0068	3	−1.3824578194*E* − 2	4.3070	0.0007
4	−1.7129433714*E* − 1	5.0145	0.0002	4	−1.3118016465*E* − 2	3.3267	0.0050
5	1.9652042724*E* − 2	3.8254	0.0021	5	−8.1160210718*E* − 3	4.6210	0.0004
6	1.9649280989*E* − 4	1.5759	0.0230	6	1.9244882179*E* − 4	1.8834	0.0120
7	1.3402240277*E* − 2	3.3195	0.0055	7	1.1153219527*E* − 3	1.9886	0.0098
8	−1.9433649876*E* − 3	1.2426	0.0430				

Significance level = 0.05.

**Table 4:** Analyses of variance and goodness of fit for the multivariate process models for sludge yields.

	Model for ln⁡(*Y*_*A*_) (7)			Model for ln⁡(*Y*_*K*_) (8)		
	DF	SS	MSS	DF	SS	MSS
Regression	8	0.9087	0.1136	7	1.4429	0.2061
Residual	13	0.0937	0.0072	14	0.1476	0.0105
Replicate	6	0.0343	0.0057	6	0.0503	0.0084
Total	21	1.0024	0.0477	21	1.5906	0.0757
Lack-of-fit	7	0.0594	0.0085	8	0.0973	0.0122

	*F* ratio		*P* value	*F* ratio		*P* value

MLR	15.7563		1.6002*E* − 5	19.5478		3.2498*E* − 6
Lack-of-fit	1.4815		0.3240	1.4506		0.3345
*R* ^2^	0.9065			0.9072		
*R*_Adjusted_^2^	0.8490			0.8608		

**Table 5:** Experimental validation of optimized extraction responses.

	Predicted sludge yield (%)	Experimental sludge yield (%)	Absolute relative error (%)
Extraction with alcohol	8.91	8.30	7.35
Extraction with ketone	3.52	3.23	9.03

## Nomenclature

*t*: Settling time, h
*T*: Operating temperature, °C
*r*: Solvent to oil ratio
*R*_Adjusted_^2^: Adjusted coefficient of determination
*R*^2^: Coefficient of determination
**X**: Design matrix
*Y*_*A*_: Sludge yield during extraction with alcohol, %
*Y*_*K*_: Sludge yield during extraction with ketone, %
${\hat{\beta }}_{K}\&{\hat{\beta }}_{A}$: Vector of the estimates of the coefficients *β*_*i*_
DF: Degree of freedom
SS: Sum of squares
MSS: Mean sum of squares.
